# Comparison of gene expression profile of the spinal cord of sprouting-capable neonatal and sprouting-incapable adult mice

**DOI:** 10.1186/s12864-019-5974-9

**Published:** 2019-07-30

**Authors:** Hiroshi Tsujioka, Toshihide Yamashita

**Affiliations:** 1 Department of Molecular Neuroscience, Graduate School of Medicine, Osaka University, Osaka, Japan; 2 WPI Immunology Frontier Research Center, Osaka University, Osaka, Japan; 3 Graduate School of Frontier Bioscience, Osaka University, Osaka, Japan; 4 Department of Neuro-Medical Science, Graduate School of Medicine, Osaka University, Osaka, Japan

**Keywords:** Axon sprouting, Pyramidotomy, Neonates, RNA-sequencing, Mouse

## Abstract

**Background:**

The regenerative ability of severed axons in the central nervous system is limited in mammals. However, after central nervous system injury, neural function is partially recovered by the formation of a compensatory neural circuit. In a mouse pyramidotomy model, axonal sprouting of the intact side of the corticospinal tract is observed in the spinal cord, and the axons make new synapses with the denervated side of propriospinal neurons. Moreover, this sprouting ability is enhanced in neonatal mice compared to that in adult mice. Myelin-associated molecules in the spinal cord or intrinsic factors in corticospinal neurons have been investigated in previous studies, but the factors that determine elevated sprouting ability in neonatal mice are not fully understood. Further, in the early phase after pyramidotomy, glial responses are observed in the spinal cord. To elucidate the basal difference in the spinal cord, we compared gene expression profiles of entire C4–7 cervical cord tissues between neonatal (injured at postnatal day 7) and adult (injured at 8 weeks of age) mice by RNA-sequencing. We also tried to identify discordant gene expression changes that might inhibit axonal sprouting in adult mice at the early phase (3 days) after pyramidotomy.

**Results:**

A comparison of neonatal and adult sham groups revealed remarkable basal differences in the spinal cord, such as active neural circuit formation, cell proliferation, the development of myelination, and an immature immune system in neonatal mice compared to that observed in adult mice. Some inflammation-related genes were selectively expressed in adult mice after pyramidotomy, implying the possibility that these genes might be related to the low sprouting ability in adult mice.

**Conclusions:**

This study provides useful information regarding the basal difference between neonatal and adult spinal cords and the possible differential response after pyramidotomy, both of which are necessary to understand why sprouting ability is increased in neonatal mice compared to that in adult mice.

**Electronic supplementary material:**

The online version of this article (10.1186/s12864-019-5974-9) contains supplementary material, which is available to authorized users.

## Background

Central nervous system injury is devastating as it frequently induces persistent neurological deficits and there is still no effective therapy to restore function besides rehabilitation. Especially, injury to the corticospinal tract (CST) causes motor deficits, because it is the main pathway involved in voluntary movement in humans [1]. The cell body of corticospinal neurons (CSNs) localize to the cerebral cortex and their axons pass through the ventral surface of the medulla oblongata (pyramid). The main CST axon crosses to the contralateral side and then passes through the lateral column, forming synapses with neurons in the spinal cord [1]. In rodents, although the main CST passes through the dorsal column of the spinal cord instead of the lateral column, the CST is essentially conserved, and it is important for precise movement [1]. For these reasons, mouse or rat CST injury models such as traumatic brain injury [2] or pyramidotomy [3] are often used to study central nervous system injury.

After central nervous system injury, truncated axons rarely regenerate in mammals. However, it is known that axonal branches from surviving neurons are newly formed and that a compensatory neural circuit is established, leading to partial functional recovery. For example, the axonal branch of the intact side of the CST grows into the denervated side of the cervical cord and forms synapses with the propriospinal neuron approximately 4 weeks after brain injury in mice [4].

Intriguingly, the sprouting ability of the CST depends on the age of the mice. In a mouse brain injury model, axonal sprouting gradually decreases from postnatal day 3 (P3) to P21 [5]. In a mouse pyramidotomy model, the sprouting ability of P7 mice is higher than that of 2-month-old mouse [6]. Previously, the absence of myelin was thought to be the cause of the enhanced sprouting ability observed in neonatal mice. Components in myelin called myelin-associated molecules are known to inhibit axonal growth [7]. In neonatal mice, myelination is not completed, and a gradual decrease in sprouting correlates with myelination [5]. However, the deletion of paired immunoglobulin-like receptor B, which is a high affinity receptor for myelin-associated molecules, does not enhance sprouting after brain injury in adult mice [5], suggesting that there are other factors that determine sprouting ability.

Intrinsic factors represent another explanation for the high sprouting ability in neonatal mice. For example, activity of the mechanistic target of rapamycin kinase pathway in CSN is increased in neonatal mice compared to that in adult mice, and deletion of phosphatase and tensin homolog, which is a negative regulator of the mechanistic target of rapamycin kinase pathway, enhances sprouting in adult mice [6]. However, sprouting ability in mutant mice is not as high as that in neonatal mice. In addition to it, pharmacological inhibition of the mechanistic target of rapamycin kinase pathway does not affect sprouting in neonatal mice after pyramidotomy [8], suggesting that there are other factors that determine this ability.

The glial response has both beneficial (such as neurite outgrowth-enhancing) and detrimental (such as axon growth-inhibiting) effects after central nervous system injury [9]. For example, expression of astroglial and inflammatory markers at the spinal cord lesion site are higher in old mice compared to young mice, and deletion of phosphatase and tensin homolog enhances axon regeneration distal to injury only in young mice [10], suggesting that altered glial response or inflammatory response affect age dependent decline of axon regenerative ability. In pyramidotomy model, microglial activation in the denervated side of the dorsal column of the spinal cord was reported 4 days after pyramidotomy in mice [11]. Therefore, it is possible that the spinal cord response in the early phase after CST injury is important for axonal sprouting, and the difference in this response might be another factor that determines the enhanced sprouting ability in neonatal mice.

Here, to elucidate the basal difference of the spinal cord between neonatal and adult mice, we compared gene expression profiles by RNA-sequencing (RNA-seq) using entire cervical cord tissues at C4-C7 levels. We also compared gene expression profile at the initial phase after pyramidotomy in an effort to identify discordant gene expression changes that inhibit axonal sprouting in adult mice. In a basal state, genes related to neurite circuit formation, cell proliferation, or the development of myelination were found to be upregulated in neonatal mice compared to expression in adult mice. A number of responses after pyramidotomy, such as the inflammatory response and axonal outgrowth, were common between neonatal and adult mice, but some genes related to the inflammatory response were selectively upregulated in adult mice. Although many transcriptomic analyses have been performed after CST injury in previous studies [12–17], none of them compared gene expression profiles between neonatal and adult spinal cords. Therefore, this is the first study to provide gene expression profiles of neonatal and adult spinal cords after pyramidotomy, which is necessary to understand why sprouting ability is enhanced in neonates.

## Results

**Fig. 1:**
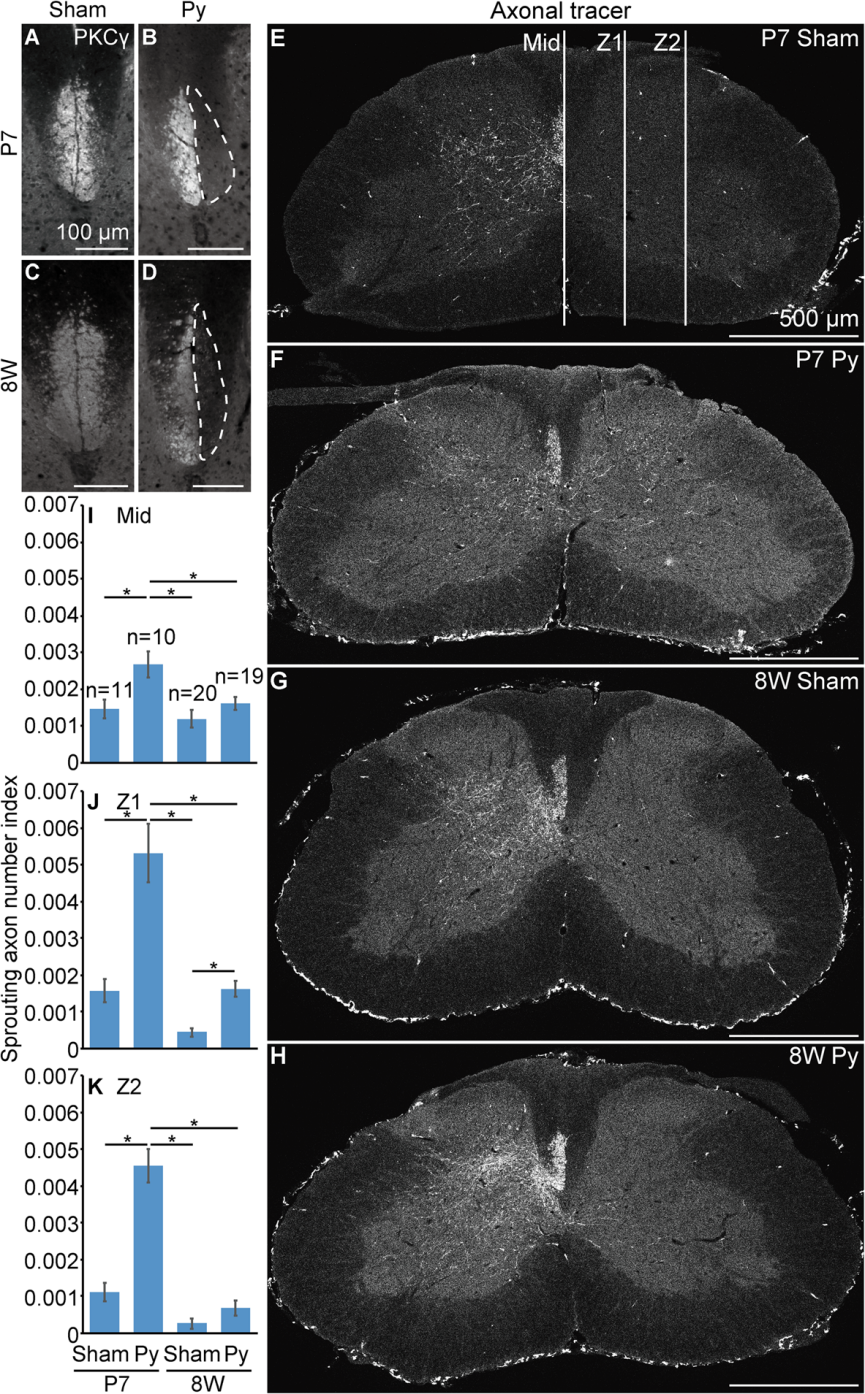
Mouse pyramidotomy model in neonates and adults. **a**-**d**, Representative images of immunohistochemistry for PKCγ (a corticospinal tract (CST) marker) using the cervical cord of mice 4 weeks after injury. The dorsal column of sham (**a**, **c**) or pyramidotomy (**b**, **d**) groups, which were injured at P7 (**a**, **b**) or 8 weeks of age (**c**, **d**), are shown. White broken lines indicate the denervated side of the CST. **e-h**, To visualize axons of the intact side of corticospinal neurons (CSNs), biotinylated or rhodamine conjugated dextran amine was injected into the intact side of CSNs and fluorescence-conjugated streptavidin or anti-rhodamine antibody following fluorescence-conjugated secondary antibody was applied to the section of the cervical cord. The axonal tracer was injected 2 weeks after injury and the mice were sacrificed 4 weeks after injury. The cervical cord of sham (**e**, **g**) or pyramidotomy (**f**, **h**) groups, which were injured at P7 (**e**, **f**) or 8 weeks of age (**g**, **h**), are shown. Signals are indicated in white. The dorsal side is at the top, ventral is bottom, left is to the left, and right is to the right. Scale bars: 100 (**a**-**d**) or 500 μm (**e**-**h**). **i-k**, Quantification of the number of sprouting axon. The number of axons crossing midline (**i**; Mid), proximal (**j**; Z1, one third from midline to the limb of gray matter) or distal part of denervated side of the gray matter (**k**; Z2, two thirds from midline to the limb of gray matter) normalized by the number of labeled axon at CST were calculated. 11 slices from 3 P7 sham mice (3–4 slices/mouse), 10 slices from 3 P7 pyramidotomised mice (3–4 slices/mouse), 20 slices from 4 8 W sham mice (3–6 slices/mouse), or 19 slices from 4 8 W pyramidotomised mice (3–6 slices/mouse) were used for quantification. Mean ± S.E.M.; *P < 0.05, Tukey’s honestly significant difference (HSD) test

First, to compare the sprouting ability between neonatal and adult mice after CST injury, we injured the left pyramid of the medulla oblongata (pyramidotomy or py) of postnatal day 7 (P7) neonatal mice or 8-week-old (8 W) adult mice. When we performed immunohistochemistry for protein kinase C gamma (PKCγ), which is a CST marker, the signal in the denervated side of the CST area of the cervical cord almost completely disappeared 4 weeks after injury in both neonatal and adult mice (Fig. 1a-d), indicating that the CST was successfully injured. Then, 2 weeks after injury, we injected anterograde tracer biotinylated or tetramethylrhodamine conjugated dextran amine into the intact side of the forelimb area of the motor cortex to label the axon of CSNs. Four weeks after injury, the number of tracer-positive axons sprouting into the denervated side of gray matter in the cervical cord was significantly higher in neonatal mice compared to that in adult mice (Fig. 1e-k), which is consistent with the results of a previous study [6].

**Fig. 2:**
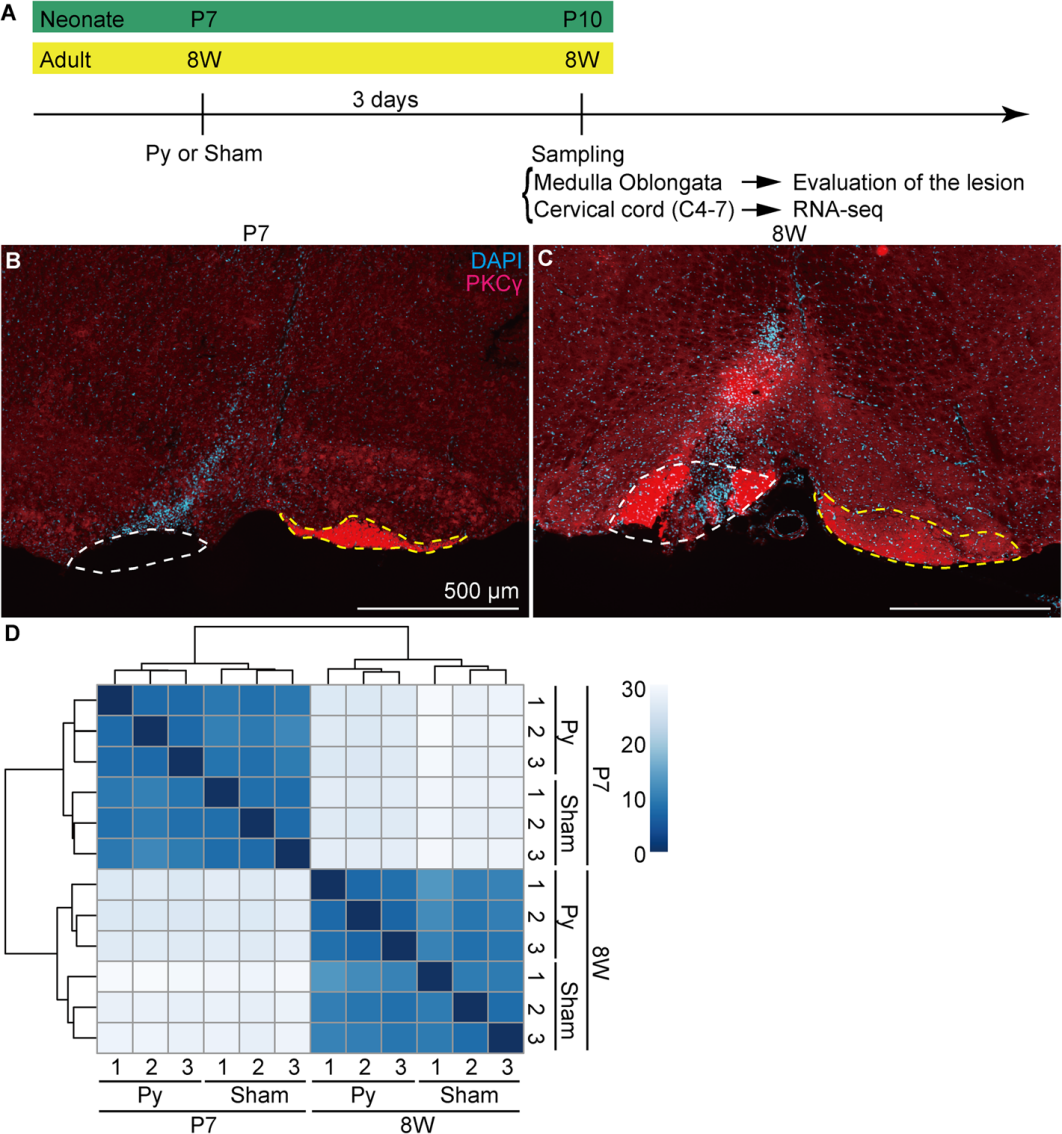
RNA-seq analysis of the neonate and adult cervical cord. **a**, Schematic drawing of the time course of sampling. **b**, **c**, Representative images of the injured site of mice selected for RNA-seq. The ventral side of the medulla oblongata of mice injured at P7 (**b**) or 8 weeks of age (**c**) are shown. Red indicates PKCγ (a corticospinal tract (CST) marker) signal and blue indicates cell nuclei stained with 4′,6-diamidino-2-phenylindole, dihydrochloride (DAPI). White and yellow broken lines indicate the injured and intact side of the CST, respectively. The dorsal side is at the top, ventral is bottom, left is to the left, and right is to the right. Scale bars: 500 μm. A negative control image of (**c**) is shown in Additional file 1. **d**, Clustering analysis using the distance matrix of RNA-seq data; *n* = 3 (lot. 1–3) in each group. The Euclidean distance between two samples is shown in corresponding colors. Blue indicates that the samples were similar (the distance is near) and white indicates that the samples were different (the distance is far)

Previously, it is reported that the microglial response in the spinal cord is observed 4 days after pyramidotomy in mice [11]. To detect early changes in the gene expression profile, we assessed the cervical cord 1 day earlier than that report (i.e. 3 days after injury (dpi)), for RNA-seq (Fig. 2a). Although single cell analysis provides useful information, cell dissociation procedure might cause artifacts. Therefore, whole tissue RNA-seq using RNA directly extracted from fresh tissue is a good choice to grasp the picture of a phenomenon as a first step. The medulla oblongata was also harvested when we collected the cervical cord and the lesion was evaluated; only animals in which a lesion was successfully generated were selected for RNA-seq analysis (Fig. 2b, c). In neonatal mice, the lesion was confirmed by a complete loss of PKCγ signal in the left CST (Fig. 2b). In adult mice, PKCγ did not disappear at 3 dpi at the lesion site (Fig. 2c). Therefore, we confirmed the successful generation of a lesion in adult mice based on the abnormally high intensity of the PKCγ signal and disrupted CST structure (Fig. 2c).

Next, 13–18 million 75-bp single-end reads were produced for each sample with three biological replicates for each group (P7 sham, P7 pyramidotomy, 8 W sham, and 8 W pyramidotomy). First, we performed cluster analysis based on a distance matrix to examine the similarity between samples. For this, each replicate in the same group clustered together (Fig. 2d) supporting the validity of RNA-seq. Moreover, samples from animals of the same age, rather than the same surgical condition, clustered together, suggesting that age has a greater effect on gene expression than surgical procedure.

**Fig. 3:**
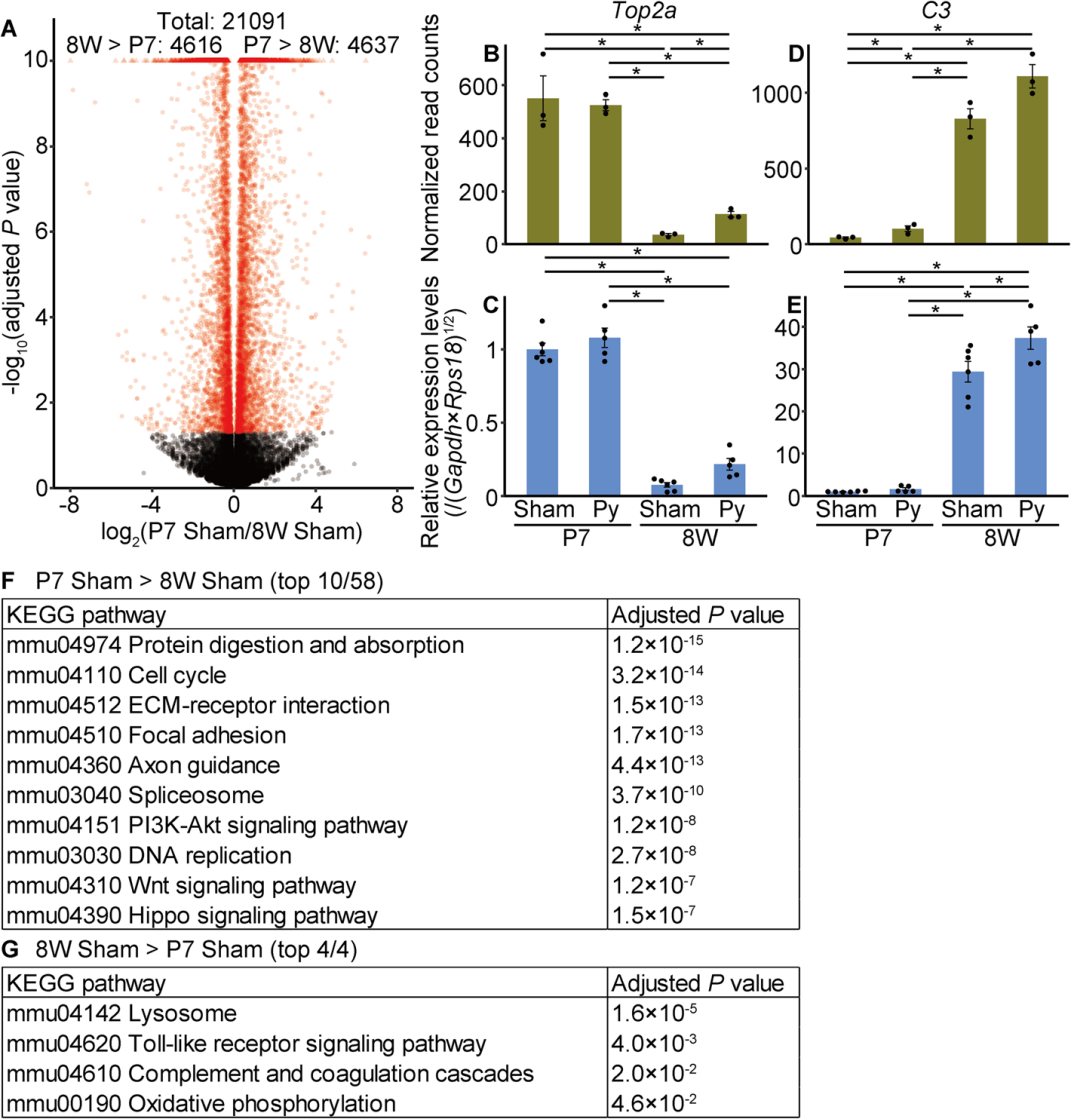
Base differences between neonatal and adult sham groups. **a**, Volcano plot. The horizontal axis represents the log_2_ fold-change in gene expression levels in the P7 sham group compared to those in the 8 W sham group, whereas the vertical axis represents the −log_10_ adjusted *P* value. Points outside the range are plotted on the edge of the graph in triangles. Genes for which the adjusted *P* was < 0.05 (Wald test) are indicated in red. **b-e**, Comparison of gene expression levels measured by RNA-seq (**b**, **d**) and qRT-PCR (**c**, **e**). The expression levels of *topoisomerase (DNA) II alpha* (*Top2a*; **b**, **c**) or *complement component 3* (*C3*; **d**, **e**) are shown. Vertical axes represent the normalized read counts (**b**, **d**) or the relative expression levels normalized to those of the geometric mean of *Gapdh* and *Rps18*, setting the value in the P7 sham group as 1 (**c**, **e**). Mean ± S.E.M.; n = 3 (**b**, **d**) or 5–6 (**c**, **e**). * adjusted *P* < 0.05, Wald test (**b**, **d**) or *P* < 0.05, Tukey’s HSD test (**c**, **e**). **f**, **g**, Pathway analyses. KEGG pathways significantly upregulated in the P7 sham group compared to those in the 8 W sham group (**f**) or those downregulated (**g**) are shown. Pathways for which the adjusted *P* was < 0.05 (two-sample *t*-test; **g**) are shown. For (**f**), the top 10 pathways among 58 significantly different pathways are shown and a complete list is presented in Additional file 4

Then, we analyzed the base change between neonatal sham and adult sham groups. For this, 4637 genes were significantly upregulated in the P7 sham group compared to expression in the 8 W sham group, and 4616 genes were significantly downregulated (Fig. 3a). We chose some of the significantly up/downregulated genes and validated the results of RNA-seq by quantitative reverse transcription-polymerase chain reaction (qRT-PCR; Fig. 3b–e; Additional file 2B–W). Results of qRT-PCR were similar to those of RNA-seq, strongly supporting the validity of RNA-seq. Gene enrichment analyses revealed that several Kyoto Encyclopedia of Genes and Genomes (KEGG) pathways such as cell cycle or axon guidance were significantly upregulated in neonatal mice (Fig. 3f, Additional file 4), whereas pathways including Toll-like receptor signaling were significantly downregulated (Fig. 3g). These results indicated a developmental change between the neonatal and adult spinal cord.

**Fig. 4:**
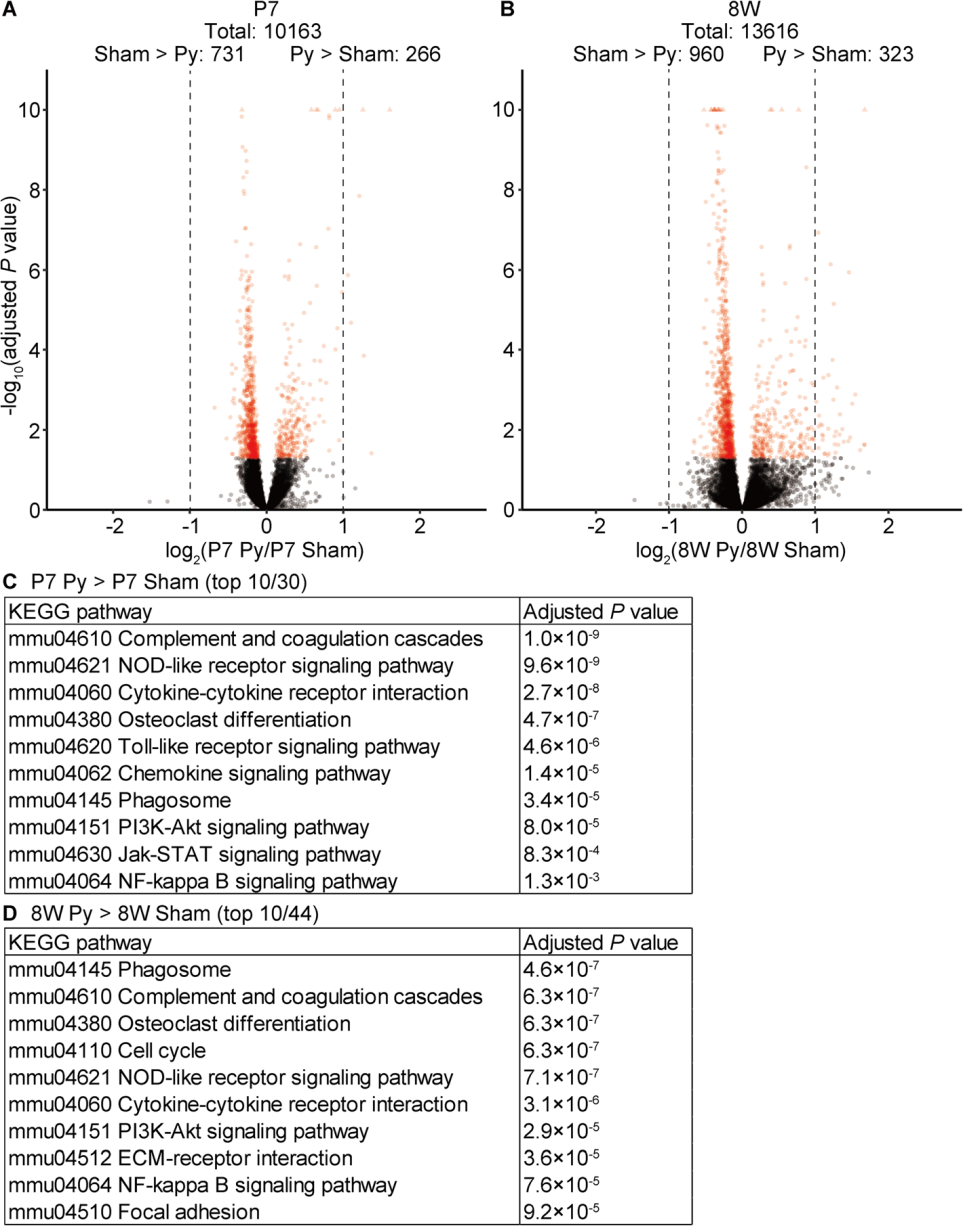
Gene expression changes after pyramidotomy in neonates and adults. **a**, **b**, Volcano plots of P7 pyramidotomy vs P7 sham (**a**) or 8 W pyramidotomy vs 8 W sham groups (**b**). Vertical axes represent the −log_10_ adjusted *P* value, whereas the horizontal axes represent the log_2_ fold-change in expression levels in the P7 pyramidotomy group compared to those in the P7 sham group (**a**) or in the 8 W pyramidotomy group compared to those in the 8 W sham group (**b**). Broken lines indicate the level of 2-fold change. Points beyond the range are plotted on the edge of the graph in triangles. Red indicates adjusted *P* < 0.05 (Wald test). Because some genes were judged as outliers and omitted from some comparisons, the total number of genes was different. **c**, **d**, KEGG pathways significantly upregulated in P7 pyramidotomy group compared to that in the P7 sham groups (**c**) or in the 8 W pyramidotomy group compared to that in the 8 W sham (**d**) group are shown. Top 10 pathways among 30 (**c**) or 44 (**d**) total pathways for which the adjusted *P* was < 0.05 (two-sample *t*-test) are shown, and the complete lists are presented in Additional files 5 and 6

We also tried to compare gene expression profiles between neonatal pyramidotomy and neonatal sham groups (Fig. 4a). Although the number of genes that were downregulated in the neonatal pyramidotomy group compared to expression in the sham group (731) was higher than the number of genes that were upregulated (266), no gene was downregulated by more than 2-fold, whereas some genes showed more than 2-fold upregulation. When we compared gene expression profiles between adult pyramidotomy and adult sham groups, a similar tendency was observed (Fig. 4b). We considered that a small fold-change might be unreliable and thus focused on pathways that were activated in the pyramidotomy group. Most of the pathways enriched in the neonatal pyramidotomy group compared to expression in the neonatal sham group (Fig. 4c) were also enriched in the adult pyramidotomy group compared to that in the adult sham group (Fig. 4d). In fact, all top 10 pathways enriched in the P7 pyramidotomy group were also significantly enriched in the 8 W pyramidotomy group (Fig. 4c, d, Additional files 5 and 6), and top 10 pathways enriched in the 8 W pyramidotomy group were also significantly enriched in the P7 pyramidotomy group (Fig. 4c, d, Additional files 5 and 6), except for the cell cycle pathway (Fig. 4d). These results suggest that the response induced by pyramidotomy is very similar between neonatal and adult mice.

**Fig. 5:**
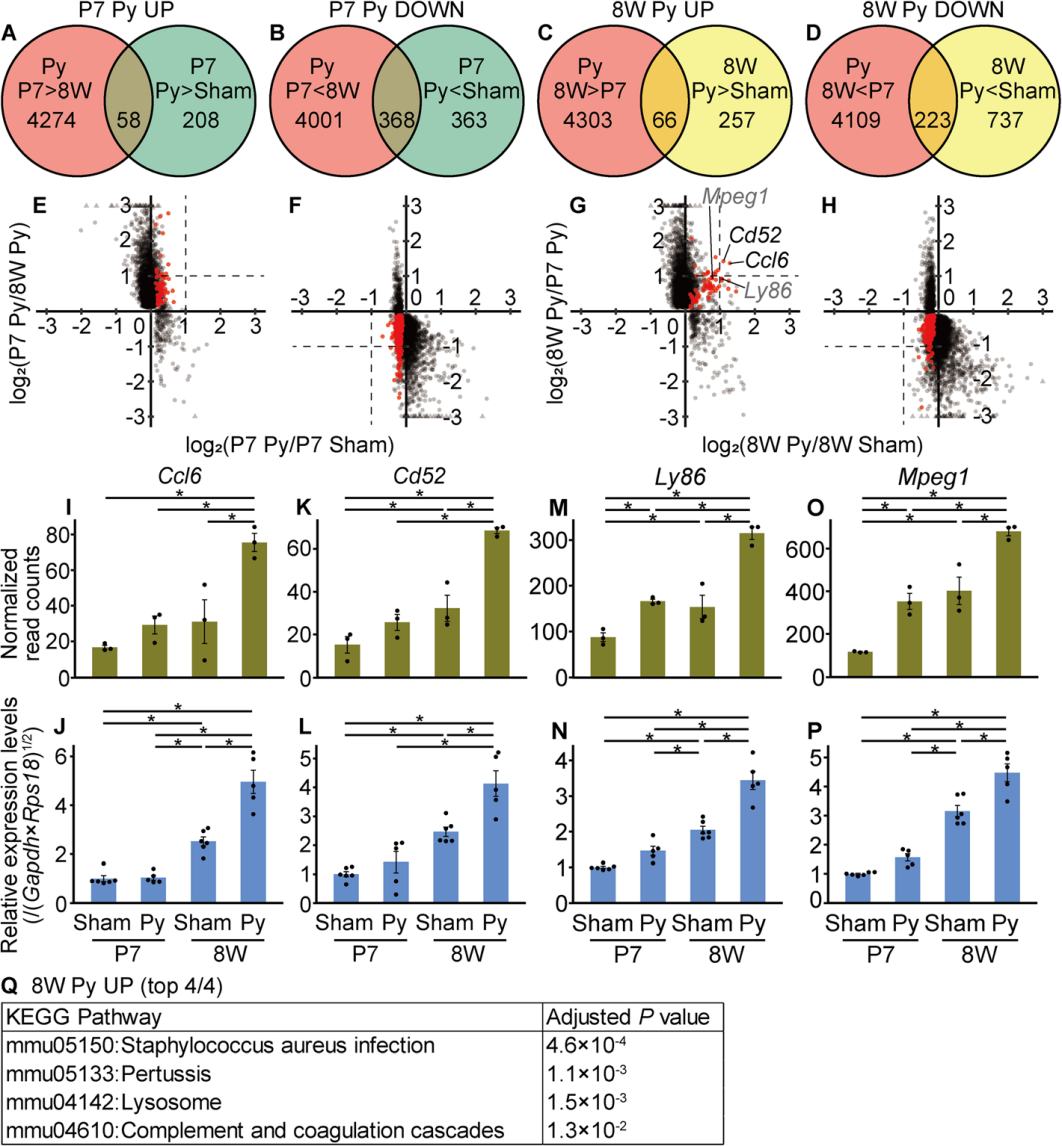
Genes selectively up/downregulated after pyramidotomy in neonates or adults. **a-d**, Venn diagrams show the number of genes significantly upregulated (**a**, **c**) or downregulated (**b**, **d**) after pyramidotomy in neonates (**a**, **b**) or adult (**c**, **d**). **e-h**, The genes shown in the Venn diagram are plotted. Red dots indicate the genes in the overlapping area of the two circles of the Venn diagrams. Horizontal axes represent the log_2_ fold-change in expression levels in the pyramidotomy group compared to those in the sham group injured at P7 (**e**, **f**) or 8 weeks of age (**g**, **h**), whereas the vertical axes represent the log_2_ fold-change in expression levels in the pyramidotomy group injured at P7 compared to those at 8 weeks of age (**e**, **f**) or injured at 8 weeks of age compared to those at P7 (**g**, **h**)). Broken lines indicate the level of 2-fold change. Points beyond the range are plotted on the edge of the graph in triangles. **i-p**, Comparison of gene expression levels measured by RNA-seq (**i**, **k**, **m**, **o**) and qRT-PCR (**j**, **l**, **n**, **p**). The expression levels of *Ccl6* (**i**, **j**), *Cd52* (**k**, **l**), *Ly86* (**m**, **n**), or *Mpeg1* (**o**, **p**) are shown. Vertical axes represent the normalized read counts (**i**, **k**, **m**, **o**) or relative expression levels normalized to those of the geometric mean of *Gapdh* and *Rps18*, setting the value in the P7 sham group as 1 (**j**, **l**, **n**, **p**). Mean ± S.E.M.; *n* = 3 (**i**, **k**, **m**, **o**) or 5–6 (**j**, **l**, **n**, **p**). * adjusted *P* < 0.05, Wald test (**i**, **k**, **m**, **o**) or *P* < 0.05, Tukey HSD test (**j**, **l**, **n**, **p**). **q**, KEGG pathways significantly (adjusted *P* < 0.05, Fisher’s exact test adjusted by Benjamini correction) enriched in the 8 W Py UP dataset are shown

In an attempt to reveal any minor differences between neonatal and adult mice after pyramidotomy, we considered both surgery and age. To identify genes that were selectively up/downregulated in neonates after pyramidotomy, we explored genes that were significantly up/downregulated in the P7 pyramidotomy group compared to expression in the P7 sham and 8 W pyramidotomy groups. Both the number of differentially expressed genes (Fig. 5a, b) and the maximum fold-change (Fig. 5e, f) were smaller for the comparison of surgery groups (P7 pyramidotomy VS P7 sham) than for the comparison of age groups (P7 pyramidotomy VS 8 W pyramidotomy), again suggesting that differences in age have a greater effect on gene expression profiles than differences in surgical procedure. When we selected some of the genes that were significantly up/downregulated based on both surgery and age comparisons (hereafter referred to as P7 Py UP/DOWN genes; red dots in Fig. 5e, f), and examined the results by qRT-PCR (Additional file 2X-AA or AB-AG), we could not detect a significant difference, possibly due to too small of differences (less than 2-fold change based on RNA-seq data). Similarly, when we explored genes significantly up/downregulated in the 8 W pyramidotomy group compared to expression in the 8 W sham and P7 pyramidotomy groups, both the number of differentially expressed genes (Fig. 5c, d) and the maximum fold-change (Fig. 5g, h) were smaller for the comparison of surgery groups (8 W pyramidotomy VS 8 W sham) than for the comparison of age groups (8 W pyramidotomy VS P7 pyramidotomy), and we could not detect significant differences by qRT-PCR for genes that were significantly downregulated based on both surgery and age comparisons (hereafter referred to as 8 W Py DOWN genes; Additional file 2AH, AI). However, the genes significantly upregulated for both surgery and age comparisons (hereafter referred to as 8 W Py UP genes) showed relatively high fold-changes. Among them, two genes (*Chemokine (C-C motif) ligand 6* (*Ccl6*) and *Cd52 antigen* (*Cd52*)) showed more than a 2-fold upregulation in the 8 W pyramidotomy groups compared to expression in the 8 W sham and P7 pyramidotomy groups. We then validated 8 W Py UP group selective expression by qRT-PCR (Fig. 5i–p) and performed gene enrichment analysis using the 8 W Py UP list (Fig. 5q). We found that KEGG pathways related to an inflammatory response (*Staphylococcus aureus* infection, pertussis, lysosome, and complement and coagulation cascade) were significantly enriched in this group. These results suggest that a specific inflammatory response is much more severe in adult mice than in neonatal mice after pyramidotomy.

**Fig. 6:**
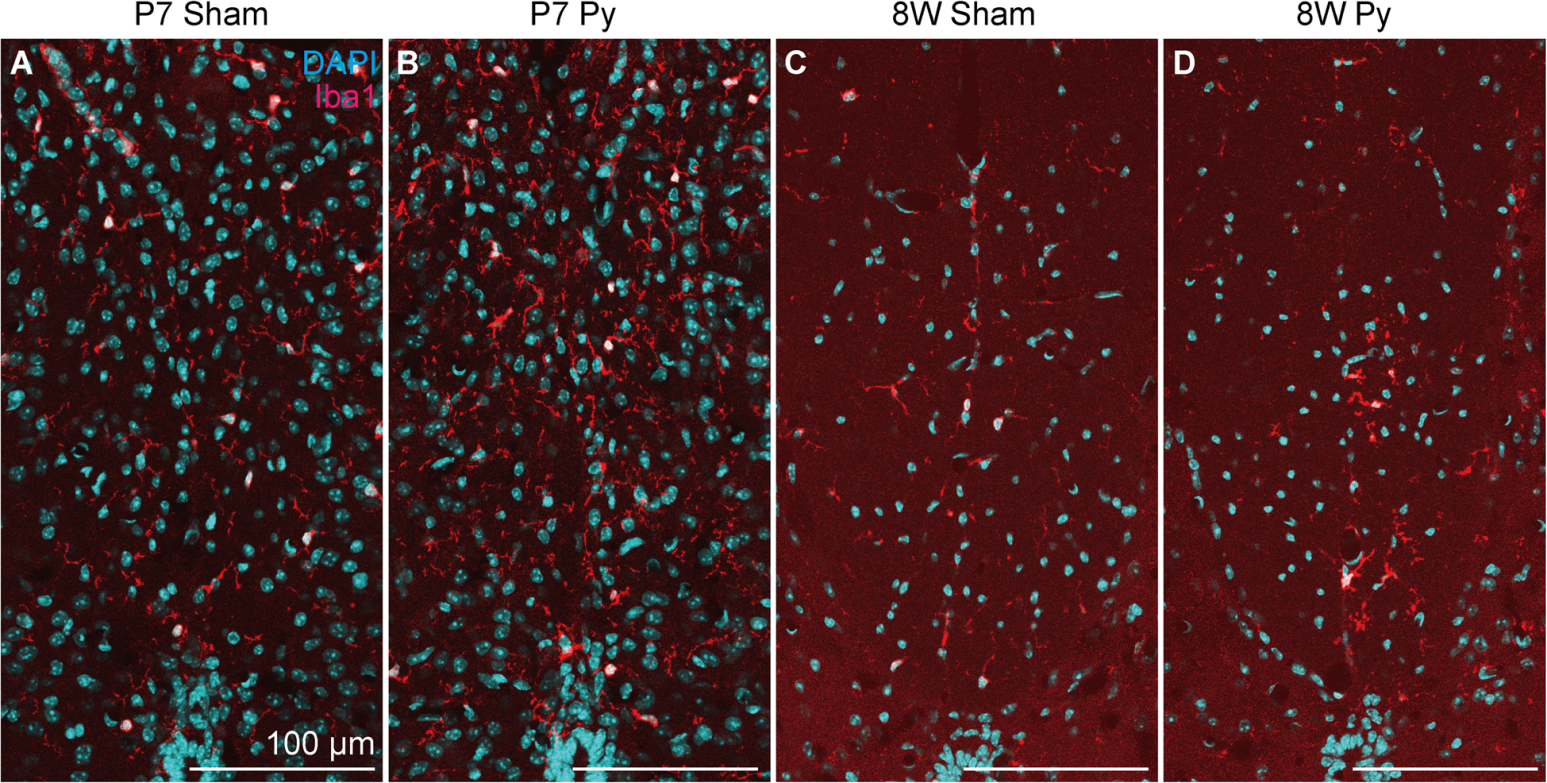
Microglial/macrophage response at the CST after pyramidotomy. Representative images of immunohistochemistry for Iba1 (a microglial/macrophage marker) using the cervical cord at 3 dpi. The dorsal column of sham (**a**, **c**) or pyramidotomy (**b**, **d**) groups, which were injured at P7 (**a**, **b**) or 8 weeks of age (**c**, **d**), are shown. Red indicates Iba1 signal, and blue indicates DAPI signal. Scale bars: 100 μm. Dorsal is at the top, ventral is bottom, left is to the left, and right is to the right

**Fig. 7:**
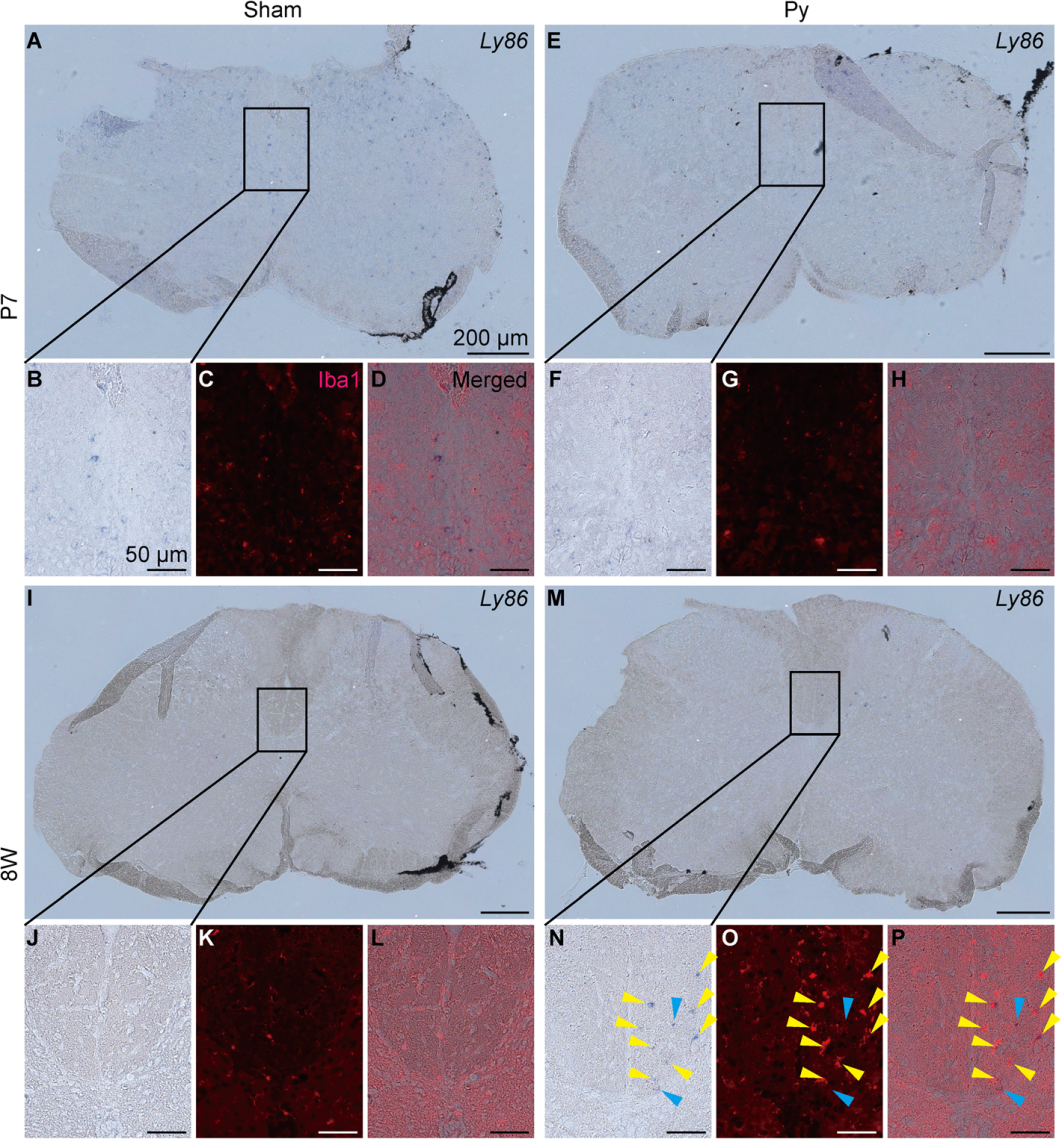
Localization of *Ly86* and microglia/macrophage. Representative images of double staining of in situ hybridization for *Ly86* and immunohistochemistry for Iba1 using the cervical cord at 3 dpi. Whole image of the spinal cord section (**a**, **e**, **i**, **m**) or magnified images of the insets (**b**-**d**, **f**-**h**, **j**-**l**, **n**-**p**) of sham (**a**-**d**, **i**-**l**) or pyramidotomy (**e**-**h**, **m**-**p**) groups, which were injured at P7 (**a**-**h**) or 8 weeks of age (**i**-**p**), are shown. Bright field (**a**, **b**, **e**, **f**, **i**, **j**, **m**, **n**), red channel (Iba1 signal, **c**, **g**, **k**, **o**), or merged images (**d**, **h**, **l**, **p**) are shown. Blue/purple indicates signal for *Ly86*. Right side of the spinal cord is marked with black pigment. Yellow arrowheads indicate colocalization of *Ly86* and Iba1, and blue arrowheads indicate *Ly86* signal which do not colocalize with Iba1 signal. Scale bars: 200 μm (**a**, **e**, **i**, **m**) or 50 μm (**b**-**d**, **f**-**h**, **j**-**l**, **n**-**p**). Dorsal is at the top, ventral is bottom, left is to the left, and right is to the right. Representative images of negative controls using sense probe are shown in Additional file 3. Because floating slices shrank during hybridization, the edge of the slices were folded in most cases when they were mounted on glass slides

**Fig. 8:**
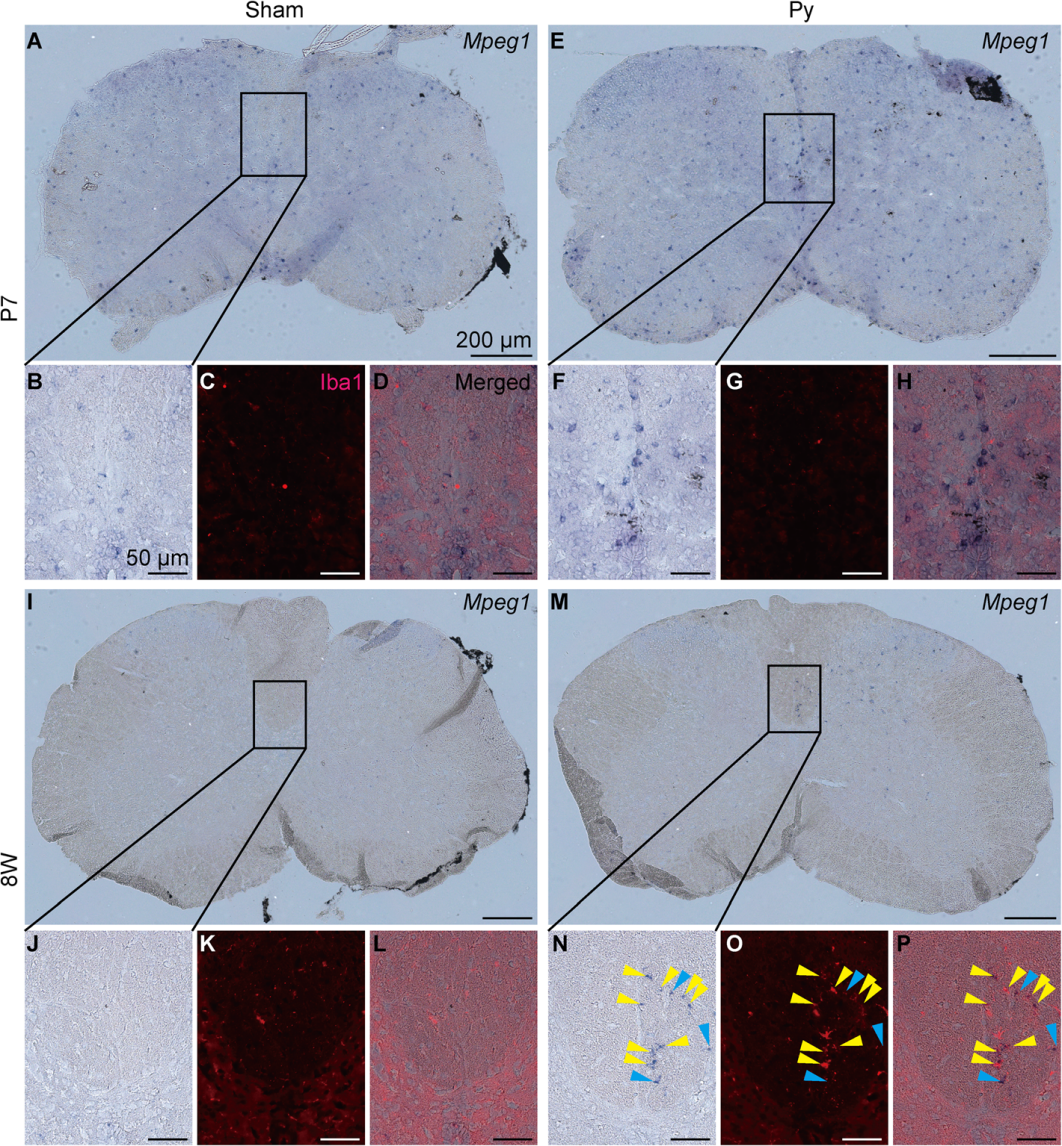
Localization of *Mpeg1* and microglia/macrophage. Representative images of double staining of in situ hybridization for *Mpeg1* and immunohistochemistry for Iba1 using the cervical cord at 3 dpi. Whole image of the spinal cord section (**a**, **e**, **i**, **m**) or magnified images of the insets (**b**-**d**, **f**-**h**, **j**-**l**, **n**-**p**) of sham (**a**-**d**, **i**-**l**) or pyramidotomy (**e**-**h**, **m**-**p**) groups, which were injured at P7 (**a**-**h**) or 8 weeks of age (**i**-**p**), are shown. Bright field (**a**, **b**, **e**, **f**, **i**, **j**, **m**, **n**), red channel (Iba1 signal, **c**, **g**, **k**, **o**), or merged images (**d**, **h**, **l**, **p**) are shown. Blue/purple indicates signal for *Mpeg1*. Right side of the spinal cord is marked with black pigment. Yellow arrowheads indicate colocalization of *Mpeg1* and Iba1, and blue arrowheads indicate *Mpeg1* signal which do not colocalize with Iba1 signal. Scale bars: 200 μm (**a**, **e**, **i**, **m**) or 50 μm (**b**-**d**, **f**-**h**, **j**-**l**, **n**-**p**). Dorsal is at the top, ventral is bottom, left is to the left, and right is to the right. Representative images of negative controls using sense probe are shown in Additional file 3. Note that whole area of the spinal cord is weakly stained with blue/purple in neonatal samples in sense probe, suggesting that the weak blue/purple signal in neonatal samples is nonspecific background. Because floating slices shrank during hybridization, the edge of the slices were folded in most cases when they were mounted on glass slides

To support this observation, we examined microglial/macrophage activation by immunohistochemistry for Ionized calcium binding adaptor molecule 1 (Iba1) at 3 dpi. In adult mice, Iba1^+^ cells accumulated at the denervated side of CST after pyramidotomy, but in neonatal mice, Iba1^+^ cells were not different between intact and injured mice (Fig. 6), suggesting that microglia/macrophage are activated at the denervated side of CST after pyramidotomy only in adult mice. To connect this observation to gene expression profile, we performed double staining of in situ hybridization for 8 W Py UP genes and immunohistochemistry for Iba1. We detected signals for *Lymphocyte antigen 86 (Ly86)* and *macrophage expressed gene 1 (Mpeg1)*, although signal intensity between neonatal and adult groups are not comparable because sensitivity is different between neonatal and adult samples. For both of the genes, in situ hybridization signal was detected at the denervated side of CST in adult mice after pyramidotomy, whereas it was not different between intact and pyramidotomy group in neonatal mice (Figs. 7, 8 and Additional file 3), which is consistent to the result of RNA-seq and qRT-PCR (Fig. 5m-p). Iba1 signal was very weak in neonatal group, possibly because proteinase treatment decreased antigenicity of Iba1 in neonatal mice. Most of the signals for *Ly86* and *Mpeg1* in the denervated side of CST in 8 W Py group colocalized with Iba1 signal (Figs. 7n-p and 8n-p), suggesting that upregulation of immune related genes in 8 W Py group is at least partially explained by microglial/macrophage activation.

## Discussion

In this study, we compared gene expression profiles between neonatal and adult mice to identify the factors that determine enhanced sprouting ability in neonatal mice. The main finding of this study is that the difference in gene expression profiles between neonatal and adult mice was very large (Figs. 2d and 3a). The top 10 pathways that were significantly enriched in neonates could be categorized into four groups as follows: (1) protein digestion and absorption, extracellular molecule (ECM)-receptor interaction, focal adhesion, axon guidance, and Hippo signaling, (2) cell cycle and DNA replication, (3) phosphoinositide-3-kinase (PI3K)–Akt and Wnt signaling, and (4) the spliceosome (Fig. 3f). Obviously, proteins are absorbed in the intestine, and not the spinal cord; probably the reason why the protein absorption pathway was detected is that many collagen-encoding genes are included in this pathway, and elevated expression of these genes led to enrichment of this pathway. Therefore, probably five pathways in the first group essentially reflect the same phenomenon, specifically, active axon elongation and neural circuit construction in neonatal mice. Adhesion acts as a molecular clutch during axonal elongation, and the growth cone moves with the aid of guidance molecules [18]. Axonal outgrowth is controlled by interactions between integrins and the ECM such as collagen, laminin, or tenascin [19]. Hippo signaling is suggested to coordinate dendrite dynamics [20]. Further, it was reported that synapse formation on Renshaw cells of the spinal cord increases until 2 weeks after birth [21, 22]. We collected the cervical cord of neonates at P10; therefore, integration of the neural circuit in the spinal cord might still be in progress at that time.

Cell cycle and DNA replication pathways might reflect active cell proliferation in the neonatal spinal cord. It has been reported that in P8 mice, approximately 10% of cells proliferate in the central canal lining of the spinal cord, whereas in adult mice, this value is less than 1% [23]. These cells are thought to differentiate into glial cells.

PI3K–Akt and Wnt signaling pathways are known to be involved in the myelination or differentiation of oligodendrocyte precursor cells [24]. A previous study from our laboratory reported that myelin can be detected in white matter as early as P8–P14 [5], suggesting that myelination is active in P10 mice used in the present study. Therefore, these pathways might reflect the differentiation of oligodendrocyte precursor cells and myelination in the neonatal spinal cord.

It was also reported that polypyrimidine tract-binding protein-associated splicing factor is highly expressed in differentiating neonatal neurons [25]. It is possible that the spliceosome is important for the full maturation of neurons in the neonatal spinal cord. In addition to this, in adult mice, the oxidative phosphorylation pathway was activated (Fig. 3g). Oxidative phosphorylation is activated when neural stem cells differentiate into mature neurons [26]. Therefore, these pathways might represent maturation of the neuron.

The other pathways enriched in adult mice were the lysosome, Toll-like receptor signaling, and complement and coagulation cascade pathways (Fig. 3g). The lysosome is an important component of phagocytic pathways in microglia [27], Toll-like receptors are important pattern recognition receptors in microglia [28], and the complement cascade is important for the inflammatory response and synapse elimination mediated by microglia [29]. In the early developmental stages, microglia have an amoeboid form and they take on a complete ramified form at P28 [30]. This suggests that the function of these cells is not completely developed in neonatal mice, and the aforementioned pathways might reflect the difference in microglial function.

Interestingly, some differentially expressed genes with prominent fold change have not been analyzed well. For example, among the genes that were significantly upregulated in the adult sham group, the fold-change of *Ethanolamine phosphate phospholyas*e (*Etnppl*) was the largest. *Etnppl* encodes a protein that catalyzes the degradation of ethanolamine phosphate [31, 32], and its expression level in the brain was found to be higher in schizophrenia and bipolar patients compared to that in the normal population [33]. This suggests that this gene is important for neural function; however, the function of *Etnppl* in vivo is not known. Interestingly, the expression level of this gene was quite low in neonates and mildly suppressed after pyramidotomy in adult mice, suggesting that its expression negatively correlates with axonal growth (Additional file 2 B, C). Analyzing this gene might reveal important aspects of neural development and neural circuit repair in future studies.

Second, we found that most differentially expressed genes after pyramidotomy were common between neonatal and adult mice (Fig. 4c, d), and among these, the top 10 pathways, except for the cell cycle pathway, were categorized into two groups as follows: (1) complement and coagulation cascades, nucleotide oligomerization domain (NOD)-like receptor signaling, cytokine-cytokine receptor interaction, osteoclast differentiation, Toll-like receptor signaling, chemokine signaling, phagosome, and nuclear factor (NF)-kappa B signaling, and (2) PI3K-Akt signaling, Janus kinase (Jak)-signal transducer and activator of transcription (STAT) signaling, ECM-receptor interaction, and focal adhesion (Fig. 4c, d). The first group comprises pathways related to the inflammatory response. Complement, pattern recognition receptors such as NOD-like or Toll-like receptors, cytokines, chemokines, and NF kappa B signaling function coordinately during the innate immune response [34], whereas osteoclast differentiation might reflect microglia/macrophage differentiation, because all of these cells are considered tissue macrophages and share the differentiation pathways [35]. The phagosome represents phagocytosis by microglia/macrophages [27]. Because these cells are known to be activated following axonal injury [36] and microglia accumulation is observed in the denervated side of the CST [11], these pathways probably reflect the activation of microglia on the degenerating CST.

PI3K, Jak–STAT signaling, ECM receptor interaction, and focal adhesion are involved in axonal sprouting or axonal growth. The deletion of phosphatase and tensin homolog, which is a negative regulator of PI3K–Akt signaling, enhances sprouting after pyramidotomy [6]. The deletion of suppressor of cytokine signaling 3, which negatively regulates the Jak–STAT pathway, also enhances sprouting after pyramidotomy [37]. These reports indicate that both pathways are important for sprouting. Axonal sprouting of the propriospinal neuron has also been suggested after spinal cord injury [38]. Considering these reports, our results implicate axonal sprouting of the propriospinal neuron in both neonates and adults after pyramidotomy.

In contrast, the cell cycle was upregulated only in adults after pyramidotomy (Fig. 4d). There are two possibilities for this: (1) in neonates, cell proliferation is already active in the basal state (Fig. 3f), which masks the activation of cell proliferation after pyramidotomy, or (2) in neonates, cell proliferation is not activated after pyramidotomy. One possible type of cell that proliferates after pyramidotomy in adult mice is ependymal cells, because after spinal cord injury, ependymal cells were previously found to proliferate [39]. Examining these possibilities and identifying proliferating cells after pyramidotomy in adult mice in future studies will deepen our understanding of the cell response after pyramidotomy and differences between neonates and adults.

Third, we found that pathways related to the inflammatory response showed significantly high expression in the adult pyramidotomy group compared to that in adult sham and neonatal pyramidotomy groups (Fig. 5q). Activation of *Staphylococcus aureus* infection and pertussis pathways probably does not mean that the mice were infected but rather reflect the inflammatory response. In the future, detailed mechanisms should be revealed not by pathway analyses, but by the analyses of individual candidate genes. Among the genes selectively expressed in the adult pyramidotomy group, only *Ccl6* and *Cd52* showed more than 2-fold upregulation (Fig. 5g, i-l). *Ccl6* is considered the rodent homolog of human *CCL23* [40, 41] and its expression is observed in some pathological conditions of the central nervous system. For example, it is expressed in neurons or infiltrating blood cells after ischemia in rats [42], or microglia/macrophages in a mouse model of experimental autoimmune encephalomyelitis [43]; moreover, CCL23 is expressed in infiltrating blood cells of the infarcted human brain [42]. CCL6 has chemoattractive activity on microglia and astrocyte [44] in rats. It is possible that in adult mice, strong expression of *Ccl6* results in microglia attraction, and that the activated microglia might suppress axonal sprouting, which is not observed in neonatal mice.

*Cd52* is expressed in lymphocytes, natural killer cells, eosinophils, macrophages, and dendritic cells [45]. It was shown that an antibody against CD52 can effectively deplete CD52-expressing cells for the treatment of multiple sclerosis (a humanized monoclonal antibody called Alemtuzumab is used) [46] or in a mouse model (experimental autoimmune encephalomyelitis) [47]. Although the function of CD52 itself is not fully understood, this protein regulates the activation of T cells [45]. Therefore, it is possible that CD52 activates microglia after pyramidotomy in adult mice.

*Ly86* and *Mpeg1* also showed adult pyramidotomy group selective upregulation in qRT-PCR, although they showed slightly less than 2-fold change in RNA-seq (Fig. 5g, m-p), and we confirmed their expression in microglia/macrophage at the denervated side of CST after pyramidotomy in adult mice by in situ hybridization (Figs. 7 and 8). *Ly86*, also known as MD-1, associates with Radioprotective 105, and is involved in lipopolysaccharide-induced B cell growth [48]. *Ly86* is selectively expressed in microglia in central nervous system [49], and its expression in macrophage is elevated by pathogen stimulation [50]. *Mpeg1*, which is a member of perforin family and also known as Perforin-2 [51], is originally identified as a macrophage specific gene [52], and its promoter is used for gene expression in macrophage [53] or microglia [54] in transgenic zebrafish. Its expression in macrophage is also upregulated after infection [55]. Therefore, upregulation of these genes might represent adult specific activation of microglia/macrophage, which was also observed in immunohistochemistry for Iba1 (Fig. 6). It is possible that the density of degenerated axons of CST is high in the dorsal column, therefore microglia/macrophage engulfing the degenerated axons is strongly activated there, and the above genes were preferentially upregulated in them.

For most cases, our RNA-seq and qRT-PCR results were very similar, but for some genes, careful interpretation is needed. We collected RNA-seq samples at Zeitgeber time 1–7, and the order of sampling was P7 pyramidotomy, P7 sham, 8 W pyramidotomy, and 8 W sham (Additional file 2A). Sampling of P7 groups were conducted on the same day, and that of 8 W groups were conducted on another same day. We found intergroup differences in the expression levels of several circadian clock genes based on RNA-seq data (Additional file 2AB-AG), and accordingly, the timing of sampling might affect the expression levels of some genes. Therefore, for qRT-PCR validation, we collected all samples at Zeitgeber time 6 (Additional file 2A) on different days, although difference of sampling date might also cause other noise. As expected, the differences in the expression levels of circadian clock genes were smaller compared to those based on RNA-seq data (Additional file 2AB-AG). Because the circadian rhythm affects many aspects of biological processes such as stem cell function [56], it is possible that other minor changes in gene expression that could not be validated by qRT-PCR might reflect differences in the timing of sampling. Nevertheless, the reproducibility of most RNA-seq data was validated by qRT-PCR, indicating that our RNA-seq data essentially reflect the true biological phenomenon.

An important limitation in this study is that we used both intact and injured side of the spinal cord for RNA-seq. Because our model is left side pyramidotomy, responses are different between the left and right sides of the spinal cord. For example, intact axons sprout into the denervated side [14], and microglia is activated only at the denervated side of CST [11]. Therefore, gene expression profile is probably different between injured and denervated sides. In a preliminary experiment, we tried to separate intact and denervated sides under a microscope, but we found that rapid and reliable separation is difficult especially for fragile and small neonatal spinal cord. Particularly, microglia activation at the denervated side of CST is observed very close to midline [11], and even a small amount of contamination of different side of CST will severely affect the result of RNA-seq. From this reason, we did not separate the intact and the denervated sides, and used both sides of the spinal cord. Including both sides of the spinal cord might be one of the reasons of the modest change of gene expression in our RNA-seq result.

Modest change of gene expression might also be due to inefficient sample selection for RNA-seq. We found that PKCγ signal did not disappear in adult mice 3 days after injury (Fig. 2c), and abnormal high intensity signal was observed at the lesion site. This is not a non-specific binding of the antibody, because we did not observe it when we used normal serum IgG as a negative control (Additional file 1). PKCγ signal at the denervated side of CST disappeared in most of the samples when we collected them 4 weeks after injury (Fig. 1d), whereas the abnormal high intensity signal was observed in most of the samples when we collected them at 3 dpi (Fig. 2c), suggesting that the signal is not derived from PKCγ in intact CST. This signal might be caused by strong binding between the antibody and partially degraded PKCγ in the injured CST. It is possible that the degenerated CST (and PKCγ) was not completely cleared by microglia yet at 3 dpi in adult mice. Because distinguishing abnormal high intensity signal and normal signal is more difficult than distinguishing no signal and normal signal, it is possible that we included imperfectly injured adult mice as RNA-seq sample, and this obscured the difference of gene expression.

Recently, sprouting neuron-specific genes have been identified by collecting cortical neurons positive for retrograde tracer injected into the denervated side of spinal cord after pyramidotomy in adult mice [16]. It would be interesting to compare their gene expression profile with that of neonatal corticospinal neuron in future studies. Combining it with our present study, our understanding about neonatal sprouting would be much deepened.

## Conclusions

In this study, remarkable difference of the gene expression profile of the spinal cord between intact neonatal and adult mice was revealed. In the neonatal spinal cord, genes related to axonal growth, cell proliferation, and myelination were upregulated, whereas those related to the immune response were downregulated compared to expression in adult mice without injury. In an attempt to reveal gene expression difference after pyramidotomy, we found that some genes involved in the inflammatory response were selectively upregulated in adult mice after pyramidotomy. Our research provides useful information about the differences between neonates and adults, and further research will reveal why sprouting ability is increased in neonates compared to that in adults.

## Methods

### Animals

C57BL/6 J wild-type female mice were obtained from the domestic company (Japan SLC). For the neonatal group, the mice were injured at P7, and for the adult group, the mice were injured at 8 weeks of age. The mice were maintained with a 12-h day 12-h night cycle and fed ad libitum in specific pathogen free condition. All surgical manipulations were performed under complete aesthesis via the intraperitoneal injection of medetomidine, midazolam, and butorphanol for adult mice or the inhalation of isoflurane for neonatal mice. The experiments were not blinded or randomized, and statistical tests were not used to pre-determine the sample size or to assess whether the data met the assumption of the approach. Health status of the animals were not examined before experiments. When the tissues were harvested, the animals were sacrificed by intraperitoneal injection of overdose of medetomidine (1.2 mg/kg, Orion Pharma), midazolam (16 mg/kg, Astellas), and butorphanol (20 mg/kg, Meiji Seika Pharma).

### Surgery

Pyramidotomy was performed essentially as described previously [3]. In brief, the ventral side of the neck was incised, the trachea and the esophagus were retracted, and an injury of ~ 0.25 mm in depth ~ 0.5 mm in width was made on the left of the basal artery. For the sham group, skin was sutured after revealing the basal artery.

### Axon tracer injection

Axon tracer injection was performed essentially as described previously [4]. In brief, 0.6 μl of 10% biotinylated (Invitrogen, D1956) or rhodamine-conjugated dextran amine (Invitrogen, D1817) in phosphate buffered saline was injected into three points of the cerebral cortex (1 mm right/0 mm anterior, 1 mm right/0.5 mm anterior, and 1.5 mm right/0.5 mm anterior to the bregma, depth = 0.6 mm, total = 1.8 μl). This area corresponds to the forelimb area of the motor cortex [57]. Injection was performed 2 weeks after pyramidotomy and mice were sacrificed 2 weeks after injection (4 weeks after pyramidotomy).

### Immunohistochemistry

Immunohistochemistry for PKCγ was performed essentially as described previously [4]. In brief, the cervical cord (vertebra C4–C7 level) was harvested after perfusion with 4% paraformaldehyde in phosphate buffer and post-fixed with the same solution for 2–6 h; alternatively, the medulla oblongata was harvested without perfusion with paraformaldehyde and post-fixed with fixative overnight. The tissues were frozen and 10–20-μm thick slices were cut and mounted on the glass slide. The specimen was incubated with 400 ng/ml of an anti-PKCγ antibody (Santa Cruz Biotechnologies, sc-211), and then incubated with secondary antibody conjugated with Alexa Fluor 488 (Invitrogen, A11008) or Alexa Fluor 568 (Invitrogen, A11011). The same concentration of IgG from normal rabbit serum (Sigma, I5006) was used as a negative control. For visualization of biotinylated dextran amine, the specimen was incubated with 4 μg/ml of fluorescence-conjugated streptavidin (Invitrogen). For enhancement of rhodamine signal, 0.1 μg/ml of an anti-rhodamine antibody (Invitrogen, A-6397) and Alexa Fluor 568 conjugated secondary antibody (A11011) were used as primary and secondary antibody, respectively, for free floating slices. Then, 1 μg/ml of 4′,6-Diamidino-2-phenylindole, dihydrochloride (DAPI) was used for nuclear staining.

For Iba1, the cervical cord was post fixed for three over nights after perfusion with paraformaldehyde. 1 μg/ml of an anti-Iba1 antibody (Wako, 019–19741) was used.

### Quantification of sprouting

Quantification of sprouting was performed essentially as described previously [6]. In brief, using an image of a coronal section of the cervical cord of a biotinylated or rhodamine-conjugated dextran amine-injected mouse, a horizontal line was drawn from the central canal to the limb of the gray matter at the denervated side, and the line was divided into three parts of equal length by two vertical lines, V1 and V2 (from medial to lateral). Then, the numbers of axons crossing V2, V1, or a vertical line at the central canal (Mid), in the gray matter were counted. The numbers were normalized by the number of axons at the main CST of the same slice. 10–20 slices from 3 to 4 mice (3–6 slices per mouse) were used in each group. The statistical analysis was performed using Tukey’s honestly significant difference (HSD) test.

### RNA-seq analysis

The cervical cord (vertebra level C4–C7) tissue was homogenized in TRIzol Reagent (Invitrogen) 3 dpi. The cervical cords of neonatal mice were harvested on the same day, and those of adult mice were harvested on the same day, but neonatal and adult samples were harvested on the different days. The homogenate was stored at − 20 °C during evaluation of the lesion by immunohistochemistry. Chloroform was added to the homogenate, and the aqueous phase was transferred to the column of an RNeasy Mini Kit (Qiagen). The aqueous phase from 1 to 3 mice were pooled, and applied to the same column. Then, RNA was extracted following the manufacturer’s protocol with on column digestion of genomic DNA using the RNase-Free DNase Set (Qiagen). The library for sequencing was constructed from total RNA using the TruSeq stranded mRNA sample prep kit (Illumina). For this, 13–18 million 75-bp single-end reads/sample were produced using HiSeq 2500 (Illumina).

The following analysis was performed essentially as described previously [58] with modifications. In brief, the reads were mapped to the mouse genome GRCm38 [59] using HISAT2 [60, 61]. Read counts were obtained using HTSeq [62] with Mus_musculus.GRCm38.91.gtf [59] as the reference gene model. The following analyses were performed using R [63] with appropriate packages. The statistical test for differentially expressed genes and clustering analysis based on the Euclidean distance matrix with variance stabilizing transformations were performed using DESeq2 [64] following the developer’s protocol. Normalized read counts were obtained using the “counts” function in DESeq2 with the option “normalized = TRUE”. This means that row read counts were normalized by size factors. Size factors indicate difference of read depth between each samples, and is obtained by calculating change of gene expression level from ideal reference (geometric mean of the samples), and take median of the value, assuming that median of the change is taken from a non-differentially expressed gene. The Wald test was used and an adjusted *P* < 0.05 was considered significant. The annotation was performed using the org. Mm.eg.db package [65]. The data were plotted on graphs using ggplot2 [66] and ggbeeswarm [67] packages. The pathway analyses were performed using gage [68] package for Figs. 3 and 4 using read count data of all genes as input or DAVID [69, 70] for Fig. 5 using the 8 W Py UP gene list as input following the developer’s protocols. For gage, a two-sample *t*-test was used and an adjusted *P* (q value) < 0.05 was considered statistically significant. For DAVID, an adjusted *P* < 0.05 based on a Fisher’s exact test adjusted using the Benjamini correction was considered statistically significant.

### qRT-PCR

RNA was extracted as described in the RNA-seq analysis section except that homogenates were not pooled, and the timing of sampling was different (Additional file 2A). Total RNA was reverse transcribed using the High Capacity cDNA Reverse Transcription Kit (Applied Biosystems). Real-time PCR was performed using Fast SYBR Green Master Mix (Applied Biosystems) and QuantStudio 7 Flex Real-Time PCR System (Applied Biosystems) following the manufacture’s protocol. cDNA samples corresponding to final RNA amount of 6–9 ng were applied to the MicroAmp Fast Optical 384-Well Reaction Plate (Applied Biosystems). The PCR condition was as follows: 95 °C for 20 s followed by 40 cycles of 95 °C for 3 s and 60 °C for 30 s. The primers used for qRT-PCR are listed in Additional file 7. The expression levels were calculated by the relative standard curve method using reverse-transcribed product from the P7 sham group or the 8 W pyramidotomy group as standards and normalized by the geometric mean of the expression levels of *glyceraldehyde-3-phosphate dehydrogenase* (*Gapdh*) and *ribosomal protein S18* (*Rps18*, [71], setting the value in the P7 sham group as 1. The Tukey’s honestly significant difference (HSD) test was used for statistical analysis with a cutoff value of *P* < 0.05.

### Double staining of in situ hybridization and immunohistochemistry

0.5–1 kbp length of in situ hybridization probes were designed for *Ly86* and *Mpeg1*. For *Mpeg1*, 5 probes targeting different region of *Mpeg1* mRNA were mixed to enhance the signal as described previously [58].in situ hybridization was performed essentially as described previously [72] with modifications. In brief, the cervical cord was fixed as described in immunohistochemistry for Iba1 section. 20 μm thick slices were cut, and the specimens were processed by free-floating method. The specimen was incubated with 0.2 μg/ml of proteinase K solution for 30 min at 37 °C following acetylation. Probe was diluted with hybridization buffer (750 mM NaCl, 75 mM sodium citrate, 50% formaldehyde, 2% blocking reagent (roche, 1096176), 0.1% 3-[(3-Cholamidopropyl) dimethylammonio] propanesulfonate, and 0.1% sodium dodecyl sulfate) at the concentration of 1 ng/μl each, and the specimen was incubated in the probe solution over night at 60 °C after pre-hybridization for 1 h. The specimen was incubated with 1:1000 dilution of alkaline phosphatase-conjugated anti-Digoxigenin antibody (roche, 11093274910) for 2-5 h after RNase treatment, following incubation with nitro blue tetrazolium chloride/5-bromo-4-chloro-3-indolyl-phosphate, toluidine-salt solution for two over nights. Then the specimen was mounted on a glass slide, and incubated with anti-Iba1 antibody solution as described in immunohistochemistry for Iba1 section after washing with methanol and ethanol.

## Additional files

Additional file 1: Negative control of immunohistochemistry for PKCγ. A representative image of negative control of Fig. 2c. Normal serum IgG was applied instead of anti-PKCγ antibody. Red indicate non-specific binding of IgG, and blue indicate DAPI signal. Dorsal is at the top, ventral is bottom, left is to the left, and right is to the right. A scale bar: 500 μm. (TIF 2989 kb)

Additional file 2: Comparison of RNA-seq and qRT-PCR. A, The tissues for RNA-seq or qRT-PCR were harvested at the indicated time points. B-AI, Comparison of the expression levels measured by RNA-seq (B, D, F, H, J, L, N, P, R, T, V, X, Z, AB, AD, AF, AH; *n* = 3) and qRT-PCR (C, E, G, I, K, M, O, Q, S, U, W, Y, AA, AC, AE, AG, AI; *n* = 5–6). Based on the RNA-seq data, genes upregulated (B-S) or downregulated (T-W) in the adult sham group compared to those in the neonatal sham group, genes in the P7 Py UP group (X-AA), P7 Py DOWN group (AB-AG), or 8 W Py DOWN group (AH, AI) were chosen. Vertical axes represent normalized read counts (for RNA-seq) or relative expression levels normalized to those of the geometric mean of *Gapdh* and *Rps18*, setting the value of the P7 sham group to 1 (for qRT-PCR). Mean ± S.E.M. * adjusted *P* < 0.05, Wald test (for RNA-seq) or *P* < 0.05, Tukey HSD test (for qRT-PCR). *Wfdc18*, *WAP four-disulfide core domain 18*; *Serpinb1a*, *serine (or cysteine) peptidase inhibitor, clade B, member 1a*; *Dao*, *D-amino acid oxidase*; *Ankub1*, *ankyrin repeat and ubiquitin domain containing 1*; *Tnfaip6*, *tumor necrosis factor alpha induced protein 6*; *Arl4d*, *ADP-ribosylation factor-like 4D*; *Kcng4*, *potassium voltage-gated channel, subfamily G, member 4*; *Ctss*, *cathepsin S*; *Eln*, *elastin*; *Sema6a*, *sema domain, transmembrane domain (TM), and cytoplasmic domain, (semaphorin) 6A*; *Col5a3*, *collagen, type V, alpha 3*; *Nes*, *nestin*; *Calca*, *calcitonin/calcitonin-related polypeptide, alpha*; *Nr1d1*, *nuclear receptor subfamily 1, group D, member 1*; *Per1*, *period circadian clock 1*; *Pou3f1*, *POU domain, class 3, transcription factor 1*. (TIF 1830 kb)

Additional file 3: Negative control of in situ hybridization for *Ly86* and *Mpeg1*. Representative images of negative controls for Figs. 7 and 8 using sense probes for *Ly86* (A-D) or *Mpeg1* (E-H) are shown. The spinal cord of sham (A, C, E, G) or pyramidotomy (B, D, F, H) group which were injured at P7 (A, B, E, F) or 8 W (C, D, G, H) are shown. Right sides of the spinal cords are marked with black pigment. Blue/purple color is non-specific binding of sense probe. Dorsal is at the top, ventral is bottom, left is to the left, right is to the right. Scale bars: 200 μm. Because floating slices shrank during hybridization, the edge of the slices were folded in most cases when they were mounted on glass slides. (TIF 9508 kb)

Additional file 4: KEGG pathways significantly upregulated in the P7 sham group compared to the 8 W sham group. KEGG pathways significantly (adjusted *P* < 0.05, two-sample *t*-test) upregulated in the P7 sham group compared to the 8 W sham group are shown. (XLSX 10 kb)

Additional file 5: KEGG pathways significantly upregulated in the P7 pyramidotomy group compared to the P7 sham group. KEGG pathways significantly (adjusted *P* < 0.05, two-sample *t*-test) upregulated in the P7 pyramidotomy group compared to the P7 sham group are shown. (XLSX 17 kb)

Additional file 6: KEGG pathways significantly upregulated in the 8 W pyramidotomy group compared to the 8 W sham group. KEGG pathways significantly (adjusted *P* < 0.05, two-sample *t*-test) upregulated in the 8 W pyramidotomy group compared to the 8 W sham group are shown. (XLSX 16 kb)

Additional file 7: List of primers used for qRT-PCR. Forward and reverse primers used for qRT-PCR are listed. (XLSX 10 kb)

## Data Availability

The RNA-seq datasets produced in this study are deposited in DNA DataBank of Japan (accession number DRA007590).

## Abbreviations

Ankub1: Ankyrin repeat and ubiquitin domain containing 1
Arl4d: ADP-ribosylation factor-like 4D
C3: Complement component 3
Calca: Calcitonin/calcitonin-related polypeptide, alpha
Ccl: Chemokine (C-C motif) ligand
Cd52: Cd52 antigen
Col5a3: Collagen, type V, alpha 3
CSN: Corticospinal neuron
CST: Corticospinal tract
Ctss: Cathepsin S
Dao: D-amino acid oxidase
DAPI: 4′,6-diamidino-2-phenylindole, dihydrochloride
dpi: Days post-injury
ECM: Extracellular molecule
Eln: Elastin
Etnppl: Ethanolamine phosphate phospholyase
Gapdh: Glyceraldehyde-3-phosphate dehydrogenase
Iba1: Ionized calcium binding adaptor molecule 1
Jak-STAT: Janus kinase–signal transducer and activator of transcription
Kcng4: Potassium voltage-gated channel, subfamily G, member 4
KEGG: Kyoto Encyclopedia of Genes and Genomes
Ly86: Lymphocyte antigen 86
Mpeg1: Macrophage expressed gene 1
Nes: Nestin
NF-kappa B: Nuclear factor kappa B
NOD-like receptor: Nucleotide oligomerization domain-like receptor
Nr1d1: Nuclear receptor subfamily 1, group D, member 1
P: Postnatal day
Per1: Period circadian clock 1
PI3K: Phosphoinositide-3-kinase
PKCγ: Protein kinase C gamma
Pou3f1: POU domain, class 3, transcription factor 1
Py: Pyramidotomy
qRT-PCR: Quantitative reverse transcription-polymerase chain reaction
RNA-seq: RNA-sequencing
Rps18: Ribosomal protein S18
Sema6a: Sema domain, transmembrane domain (TM), and cytoplasmic domain, (semaphorin) 6A
Serpinb1a: Serine (or cysteine) peptidase inhibitor, clade B, member 1a
Tnfaip6: Tumor necrosis factor alpha induced protein 6
Top2a: Topoisomerase (DNA) II alpha
Tukey’s HSD test: Tukey’s honestly significant difference test
W: Weeks old
Wfdc18: WAP four-disulfide core domain 18
