# Supplementary figures and images for: Comparison of gene expression profile of the spinal cord of sprouting-capable neonatal and sprouting-incapable adult mice
Source: BMC Genomics. 2019 Jul 30;20:619. doi: 10.1186/s12864-019-5974-9 (PMC6668129; doi:10.1186/s12864-019-5974-9)

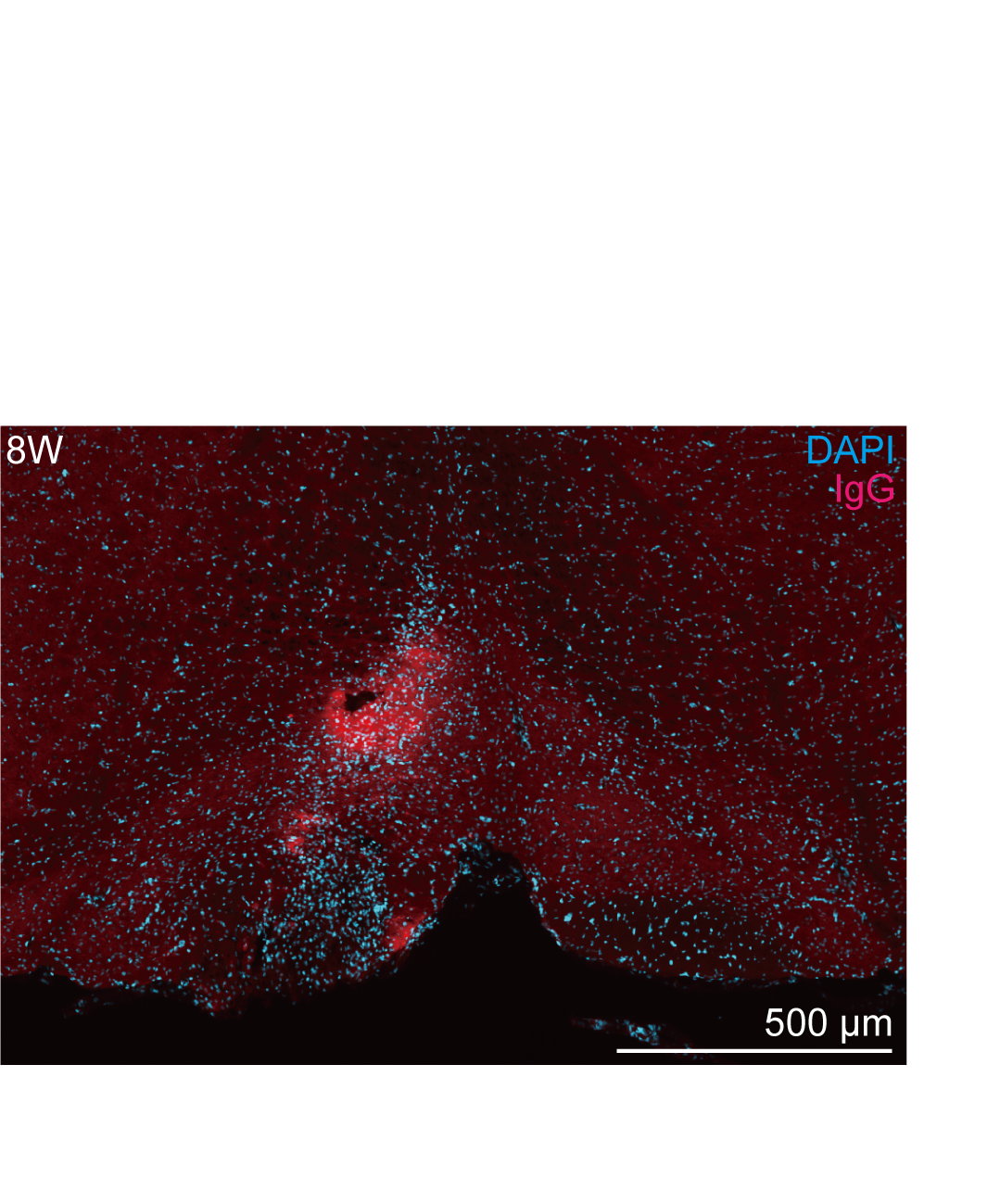

Supplement: Supplementary file 1 — Negative control of immunohistochemistry for PKCγ. A representative image of negative control of Fig. 2c. Normal serum IgG was applied instead of anti-PKCγ antibody. Red indicate non-specific binding of IgG, and blue indicate DAPI signal. Dorsal is at the top, ventral is bottom, left is to the left, and right is to the right. A scale bar: 500 μm. (TIF 2989 kb) [file 12864_2019_5974_MOESM1_ESM.tif]

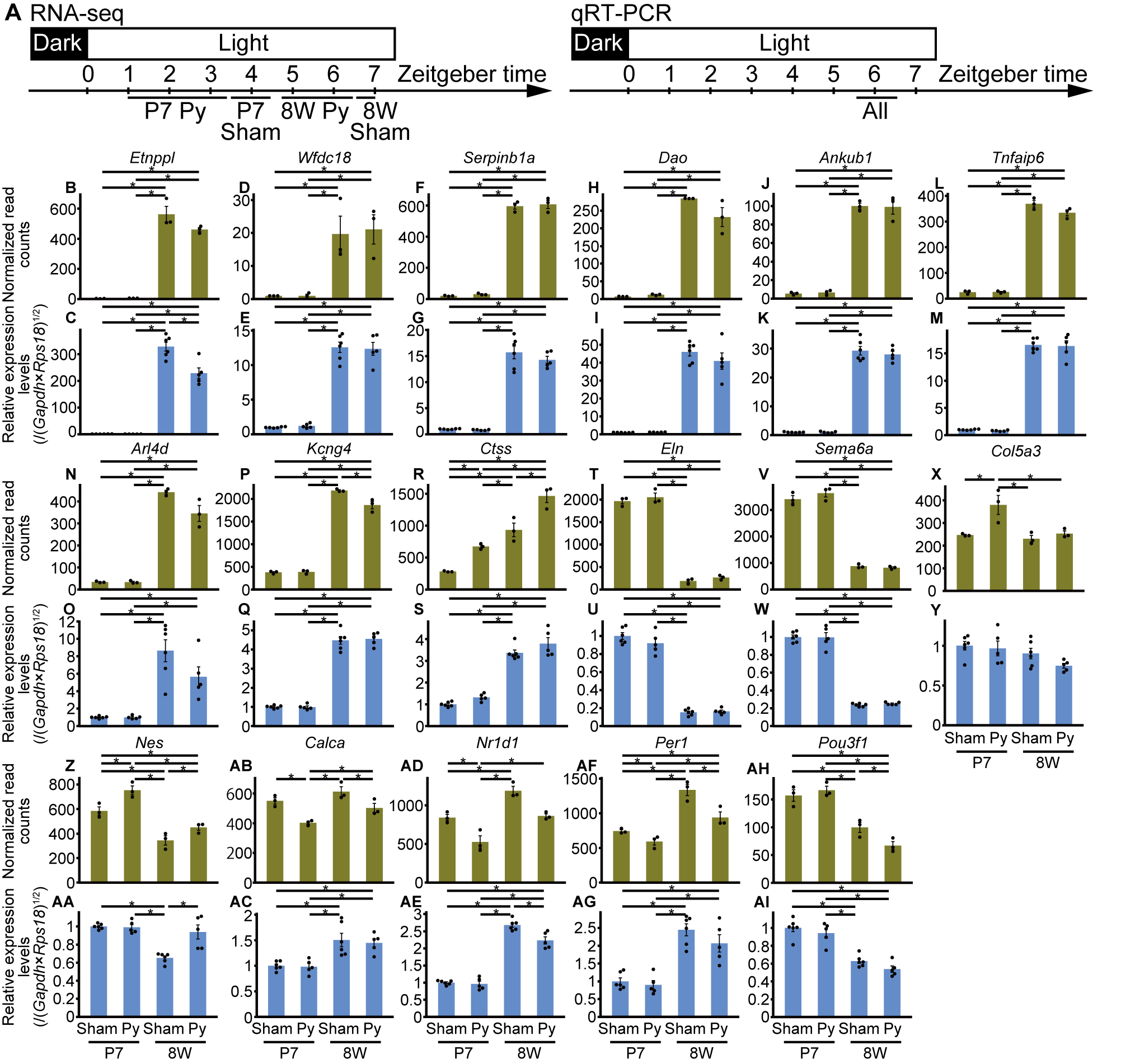

Supplement: Supplementary file 2 — Comparison of RNA-seq and qRT-PCR. A, The tissues for RNA-seq or qRT-PCR were harvested at the indicated time points. B-AI, Comparison of the expression levels measured by RNA-seq (B, D, F, H, J, L, N, P, R, T, V, X, Z, AB, AD, AF, AH; n = 3) and qRT-PCR (C, E, G, I, K, M, O, Q, S, U, W, Y, AA, AC, AE, AG, AI; n = 5–6). Based on the RNA-seq data, genes upregulated (B-S) or downregulated (T-W) in the adult sham group compared to those in the neonatal sham group, genes in the P7 Py UP group (X-AA), P7 Py DOWN group (AB-AG), or 8 W Py DOWN group (AH, AI) were chosen. Vertical axes represent normalized read counts (for RNA-seq) or relative expression levels normalized to those of the geometric mean of Gapdh and Rps18, setting the value of the P7 sham group to 1 (for qRT-PCR). Mean ± S.E.M. * adjusted P < 0.05, Wald test (for RNA-seq) or P < 0.05, Tukey HSD test (for qRT-PCR). Wfdc18, WAP four-disulfide core domain 18; Serpinb1a, serine (or cysteine) peptidase inhibitor, clade B, member 1a; Dao, D-amino acid oxidase; Ankub1, ankyrin repeat and ubiquitin domain containing 1; Tnfaip6, tumor necrosis factor alpha induced protein 6; Arl4d, ADP-ribosylation factor-like 4D; Kcng4, potassium voltage-gated channel, subfamily G, member 4; Ctss, cathepsin S; Eln, elastin; Sema6a, sema domain, transmembrane domain (TM), and cytoplasmic domain, (semaphorin) 6A; Col5a3, collagen, type V, alpha 3; Nes, nestin; Calca, calcitonin/calcitonin-related polypeptide, alpha; Nr1d1, nuclear receptor subfamily 1, group D, member 1; Per1, period circadian clock 1; Pou3f1, POU domain, class 3, transcription factor 1. (TIF 1830 kb) [file 12864_2019_5974_MOESM2_ESM.tif]

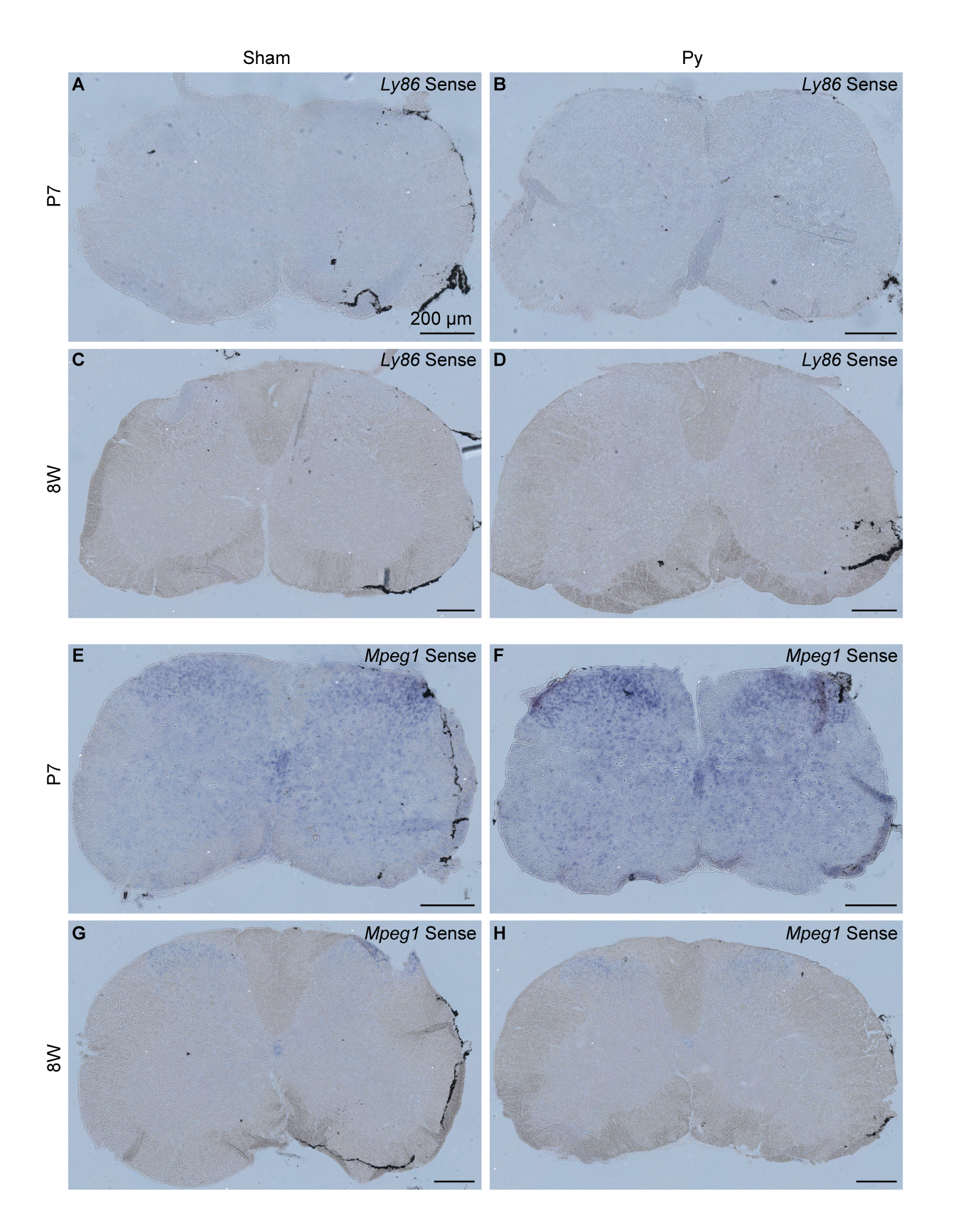

Supplement: Supplementary file 3 — Negative control of in situ hybridization for Ly86 and Mpeg1. Representative images of negative controls for Figs. 7 and 8 using sense probes for Ly86 (A-D) or Mpeg1 (E-H) are shown. The spinal cord of sham (A, C, E, G) or pyramidotomy (B, D, F, H) group which were injured at P7 (A, B, E, F) or 8 W (C, D, G, H) are shown. Right sides of the spinal cords are marked with black pigment. Blue/purple color is non-specific binding of sense probe. Dorsal is at the top, ventral is bottom, left is to the left, right is to the right. Scale bars: 200 μm. Because floating slices shrank during hybridization, the edge of the slices were folded in most cases when they were mounted on glass slides. (TIF 9508 kb) [file 12864_2019_5974_MOESM3_ESM.tif]
